# Changes in excessive alcohol use among older women across the menopausal transition: a longitudinal analysis of the Study of Women’s Health Across the Nation

**DOI:** 10.1186/s13293-020-00314-7

**Published:** 2020-07-14

**Authors:** MacKenzie R. Peltier, Terril L. Verplaetse, Walter Roberts, Kelly Moore, Catherine Burke, Phillip L. Marotta, Sarah Phillips, Philip H. Smith, Sherry A. McKee

**Affiliations:** 1 Department of Psychiatry, Yale School of Medicine, 2 Church Street South, Suite 201, New Haven, CT 06519 USA; 2 Psychology Service, VACT Healthcare System, West Haven, CT 06510 USA

**Keywords:** Menopause, Women, Excessive drinking, Alcohol use disorders, Menopausal transition

## Abstract

**Background:**

Recent data suggest that excessive alcohol use is increasing among women and older adults. Such trends are concerning, as women are more vulnerable to alcohol-related health consequences, and such health problems may be exacerbated with age. Furthermore, there are sex-specific factors that may influence alcohol consumption among women, including the hormonal changes associated with the menopausal transition and negative affect. The present study sought to investigate transitions in excessive drinking among women across the menopausal transition and included exploration of sex hormones (estradiol; testosterone) and depression.

**Methods:**

The present study utilized publicly available data from the Study of Women Across the Nation (SWAN) and included 3302 women (42–52 years old at baseline), who completed 10 years of annual assessments. National Institute on Alcohol Abuse and Alcoholism (NIAAA) criteria were used as guidance when defining excessive drinking within the present dataset. At year 1, 170 women were identified as drinking excessively. Random-effect logistic regressions were used to examine transitions in excessive drinking.

**Results:**

Women identified as excessive drinkers were more likely to transition to non-excessive drinking across all menopausal transition stages (*ORs* range = 3.71–5.11), while women were more likely to transition from non-excessive to excessive drinking during the early peri- and postmenopausal stages (*OR* = 1.52 and 1.98, respectively). Higher testosterone levels were associated with a decreased likelihood of transitioning to non-excessive drinking (*OR* = 0.59). Depression and estradiol levels were not related to transitions in drinking.

**Conclusions:**

The present study demonstrates that the menopausal transition marks a period of instability in alcohol use among women. Further research is warranted to understand factors related to transitioning in and out of excessive drinking.

## Background

Historically, alcohol use disorders (AUDs) have been observed in greater rates among males; however, recent data demonstrate that there has been an increase in excessive drinking among women [1]. In fact, over the past 10 years, rates of AUDs have increased by 84% in women, relative to a 35% increase observed in men [2]. Similarly, substantial increases have been observed in AUD among older adults, with a 35% increase among adults 45–65 years old and a 58% increase among individuals over 65 years old [2]. Such increases in excessive alcohol use are concerning, given that women experience greater alcohol-related health consequences than their male counterparts [3], and these health consequences may be exacerbated as women age [4, 5]. Furthermore, data spanning 1999–2017 demonstrated the largest increase in alcohol-related deaths annually was among non-Hispanic white women, and additional increases were observed among individuals 55–64 years old [6]. However, to date, limited research has explored alcohol consumption among older women, especially in regard to variables that may uniquely influence women’s drinking patterns, including the hormonal changes associated with the menopausal transition and negative affect (such as depressive symptoms) [7].

Recent evidence suggests that alcohol use affects reproductive function, menstrual cyclicity, and hormonal levels across the lifespan, including in postmenopausal women [8, 9]. During the menopausal transition, estradiol and progesterone initially fluctuate, but then gradually decrease to low levels of postmenopause. To date, there have been observed associations between alcohol consumption and increases in endogenous estradiol in postmenopausal women, via alcohol-related increases in aromatization and decreased estradiol metabolism [4, 10]. It has been suggested that low-risk alcohol consumption among women across the menopausal transition may, in fact, be protective against many symptoms and conditions associated with menopause; however, as the amount of alcohol consumed increases, alcohol is not increasingly protective and may place women at heightened risk for negative health consequences [4]. While testosterone has been associated with increased drinking in both sexes [10], there are minimal changes in testosterone throughout the menopausal transition [11], suggesting that testosterone may not differentially contribute to changes in drinking during this time. Given the hormonal changes observed during the menopausal transition, it is important to explore the role of such changes in alcohol consumption among this population of women especially considering their increased risk of health consequences.

In addition to the hormonal changes associated with the menopausal transition, another important factor in women’s alcohol consumption is negative affect, as women are generally more likely to drink for negative reinforcement (e.g., drinking to regulate stress, negative mood, and negative affect, including depression) [7]. While one study has demonstrated that alcohol use is not strongly associated with depression across 20 years among women over the age of 65 [12], there is scant literature exploring the relationship between alcohol and depression during menopause. Menopause has been categorized as a time of increased negative affect, including psychosocial stress and depression, among other symptoms of psychological impairment [13], which may lead older women to drink for negative reinforcement of such affect. Additionally, among older adults, “reactive” drinking may be excessive, in that as psychosocial stressors connected to later life stages (e.g., retirement and widowing) emerge, and they are more likely to regularly consume alcohol [4]. However, there is some mixed evidence indicating that happiness occurs in a “U-Shape” across the lifespan, with an increase in contentment occurring initially in young adulthood and then again in “old age” (for review, see [14]).This warrants additional research into the connection between negative affect and alcohol use in older women.

## Method

The present study sought to investigate the effect of the menopausal transition, as well as the effect of sex hormones (estradiol and testosterone) and negative affect (depressive symptoms), on excessive drinking patterns utilizing publicly available data from the Study of Women Across the Nation (SWAN). Unfortunately, progesterone levels were not included as part of the publicly available dataset and thus were unable to be included in the present analyses. Overall, it was hypothesized that the stages of late peri-menopause and postmenopause would be associated with an increased likelihood of transition to quitting excessive drinking and a decreased likelihood of onset of excessive drinking patterns. It was hypothesized that increasing levels of estradiol, as well as increased self-reported depressive symptoms, would be related to a decreased likelihood of transitioning from excessive to non-excessive drinking behaviors and increased likelihood of transitioning from non-excessive to excessive drinking behaviors. It was hypothesized that testosterone would be unrelated to transitions in excessive drinking behaviors across the menopausal transition.

### Data source

The present study utilized publicly available data from the nationally representative SWAN dataset. SWAN enrolled 3302 women aged 42–52 years old from 1996 to 1997 across seven, geographically representative locations across the USA. All women enrolled in SWAN reported being premenopausal or early perimenopausal (defined as “reported a menstrual period within the past 3 months”), denied taking hormonal medications within the past 3 months, and similarly denied history of total hysterectomies at baseline. SWAN participants in the publicly available dataset were followed annually after completing the baseline visit. Participants attended in-person examinations which assessed for a variety of biopsychosocial variables via both survey and biological measurements (e.g., hormone levels) each year [15].

### Statistical analysis

SWAN datasets including each year’s data were merged, and all variables were matched based upon a SWAN-derived identification variable. Participants’ stage in the menopausal transition (*pre-, early peri-, late peri-, and postmenopausal*) was identified in each annual dataset. For the purpose of the current analyses, once categorized, participants were not able to be categorized “backwards” in subsequent years. Specifically, women were identified as *premenopausal* if they reported regularly occurring menses within the past 3 months. *Early peri-menopause* was categorized as women who had menses within the past 3 months, but menses did not occur regularly. Women who endorsed no menses for the past 3–11 months were categorized as *late peri-menopausal*, whereas women with no menses for over the past 12 months were identified as *postmenopausal.* Of note, women taking hormonal therapies, as well as those who endorsed having hysterectomies/ovariectomies over the course of the study, were identified as “unknown”, and their data was not included for the particular year. Finally, in years during which women reported pregnancy and/or breastfeeding, women were excluded from the present analyses.

The current analyses only included participants who transitioned to postmenopausal status over the course of the 10 years of publicly available data. A dichotomous variable for excessive alcohol consumption was derived using the data that was publicly available within the dataset, to represent individuals who drank more than 30 drinks per month (yes vs. no) from variables identifying the amount of beer, wine, and liquor consumed per past month. Only information on drinking quantity was available. This dichotomous variable was created following guidance from the National Institute on Alcohol Abuse and Alcoholism (NIAAA) criteria for hazardous drinking in women (exceeding 7 drinks per week) [16]. Excessive drinkers were identified at visit 1 (1 year following baseline visit), and then transitions were derived from annual assessment of excessive drinking behavior. Of note, variables assessing drinking quantity were not administered at baseline or year 5 and 9 visits; therefore, these results do not include data from those years.

Random-effects logistic regression models with robust standard errors were used to examine transitions in excessive drinking behavior (e.g., transition from excessive drinking to non-excessive drinking). Specifically, the first model examined the transition from excessive to non-excessive drinking, and the second model explored the transition from non-excessive drinking to excessive drinking. Data was not censored at transition from/to excessive drinking in order to best capture transitions across the 10-year period. All models included baseline age (in years), age at postmenopause, and length of menopausal transition (from *early peri- to postmenopausal status*). Additionally, measures of estradiol and testosterone, which were assessed annually, were included in the two models. These hormonal assay results did not have normal distributions and thus, in accordance with previously published SWAN hormonal data results [17–19], natural log transformations were used. Again, progesterone levels were not included within the publicly available dataset and thus could not be included in the present analyses. Finally, depressive symptoms were assessed each year, via the Center of Epidemiological Studies- Depression (CES-D) survey. These results were also included in the abovementioned models.

## Results

### Demographics

Women (*n* = 3302) had a mean age of 45.85 years old (SD = 2.69) at baseline. The majority of the women included in SWAN were Caucasian (47.0%; 28.3% Black/African American; 16.1% identified as Chinese/Japanese descent; 8.6% Hispanic). At visit 1, 170 women were identified as excessive drinkers, as they reported consuming over 30 drinks per month. Similarly at visit 10, 166 women were identified as excessive drinkers using this same criteria.

### Transition from excessive to non-excessive drinking

Women were 3–5 times more likely (*ORs* range = 3.71–5.11) to transition to non-excessive drinking during the early/late peri- and postmenopausal stages, when compared to pre-menopausal stage. Additionally, increased age at postmenopause status categorization was associated with a greater likelihood to transition to non-excessive drinking, whereas higher testosterone levels were associated with a decrease in likelihood of transition to consuming less than 30 drinks per month. There were no significant associations between estradiol levels and depressive symptoms and this transition (See Table 1).

**Table 1:** Association between menopausal status and transition from excessive to non-excessive drinking

	Hazard ratio	95% CI
Menopausal status	REF = premenopausal	
Early peri-menopausal	3.81***	2.40–6.03
Late peri-menopausal	5.11***	2.81–9.30
Postmenopausal	3.71***	2.00–6.90
Age at baseline (years)	0.92	0.83–1.02
Age at postmenopausal status	1.15*	1.02–1.30
Length on transition^a^	0.92	0.82–1.02
Estradiol (pg/mL)^b^	1.17	0.92–1.49
Testosterone (ng/dL) ^b^	0.59**	0.40–0.87
Depression (CES-D)	1.01	0.92–1.03

^a^Length from early peri- to postmenopausal status

^b^Natural log transformed

*p ≤ 0.05; **p ≤ 0.01; ***p ≤ 0.001

### Transition from non-excessive to excessive drinking

Conversely, women in the early peri- and postmenopausal stages were more likely (*OR* = 1.52 and 1.98, respectively) to transition to excessive drinking, as compared to the pre-menopausal stage. Being older at baseline and having a longer length of the transition were associated with a decreased likelihood of transitioning to excessive drinking. Sex hormones and depression were not associated with changes to excessive drinking patterns (See Table 2).

**Table 2:** Association between menopausal status and transition from non-excessive to excessive drinking

	Hazard Ratio	95% CI
Menopausal status	REF = premenopausal	
Early peri-menopausal	1.52**	1.12–2.07
Late peri-menopausal	1.16	0.68–1.98
Postmenopausal	1.98***	1.45–2.71
Age at baseline (years)	0.88*	0.79–0.98
Age at postmenopausal status	1.13**	1.03–1.25
Length on transition^a^	0.88*	0.81–0.95
Estradiol (pg/mL)^b^	0.98	0.80–1.21
Testosterone (ng/dL) ^b^	0.91	0.79–1.06
Depression (CES-D)	1.01	0.98–1.04

^a^Length from early peri- to postmenopausal status

^b^Natural log transformed

*p ≤ 0.05; **p ≤ 0.01; ***p ≤ 0.001

## Discussion

The present results demonstrate that women, who were identified as excessive drinkers, were more likely to transition to non-excessive drinking status during all three stages of the menopausal transition when compared to pre-menopause, while women who were identified as non-excessive drinkers were more likely to transition to excessive drinking during early peri- and postmenopausal transition stages. Given that there was little difference between the number of women identified as excessive drinkers between visit 1 and visit 10 (*n* = 170 and *n* = 166, respectively), this represents shifts among the women who are excessively drinking as they age. These results replicate previous research findings that alcohol use is related to onset of menopause and the menopausal transition. The present study demonstrated that increased age at postmenopausal status was associated with a 15% increase in the likelihood of transitioning to non-excessive drinking, and there was a 13% increase in the likelihood of those transitioning to excessive drinking. Being older at baseline and having a longer length of menopausal transition were also associated with a decreased likelihood of being categorized as an excessive drinker later in the study. These results illustrate the connection between excessive alcohol consumption and the menopausal transition. Previous literature has established that heavy or moderate drinking patterns are related to later onset of menopause; however, additional research is warranted to explore if later onset of menopause is directly attributable to alcohol consumption, or is representative of other biopsychosocial variables, such as demographics [20].

Surprisingly, depressive symptoms were not related to transitions in drinking status in the present study. This finding is inconsistent with the large body of evidence that women are more likely to drink to regulate negative affect, including depressive symptoms; however, it is consistent with the existing evidence that happiness may increase as one ages [14]. However, the SWAN dataset only evaluated past week depressive symptoms at one time point annually in the present study. Thus, this may not be an accurate representative of the fluctuations in affect observed across the menopausal transition and warrants further investigation. Furthermore, while higher testosterone levels were associated with a decreased likelihood to transition to non-excessive drinking, testosterone levels were not associated with transitions to heavier alcohol consumption. Estradiol was not associated with any changes in drinking. While previous research has demonstrated associations between increased levels of testosterone and estradiol and increased risk of alcohol consumption and AUDs, such results have generally been observed to be sex-specific. The association between alcohol and testosterone is generally stronger among males, and the relationship between alcohol and estrogens is generally stronger among women. Further, given the limited literature on sex hormones and alcohol use among clinical samples to date, a recent systematic review suggested comprehensive hormonal assays be utilized to explore the impact of sex hormones on alcohol use [10]. Accordingly, the present study’s use of an annual hormonal assay at a single time point may not fully capture the relationship between sex hormones and alcohol, especially given the dynamic nature of these hormones during the menopausal transition [21].

Such results are promising in that older women who drink excessively are more likely to decrease their drinking across menopause. However, we also identified that some women are newly identified as excessive drinkers, corroborating recent findings documenting increased excessive drinking in groups of women and older adults [1, 2].

Regarding women transitioning to non-excessive drinking, additional research is warranted to explore the etiology of decreased alcohol consumption. For instance, given that women are more sensitive to developing health-related problems as a result of heavy alcohol consumption, including higher risk of developing alcohol-related liver injury, cancer, and cardiovascular conditions [7] and experiencing such health consequences earlier in life [13], these findings may be indicative that these women are intersecting with the medical community more frequently and earlier on for alcohol-related conditions. While the present study cannot infer such relationships, previous research demonstrates that women are more likely to seek medical care than their male counterparts, thus illustrating an important opportunity for screening and detection of excessive alcohol consumption [4]. Motivational interviewing is widely implemented in primary care and other medical settings, and women with alcohol-related illnesses may be encouraged to reflect on drinking patterns and make behavioral changes, especially since older women are less likely to seek treatment for alcohol use in specialized treatment settings [5]. This is further supported by evidence suggesting the assessment of smoking behaviors and health (e.g., lung age and blood pressure) was related to a decrease in cigarettes per day in a previous study of postmenopausal women [22]. Thus, such intersection with medical providers may be beneficial in assisting older women in cutting down or stopping alcohol use. However, additional screening tools and therapies (including psychotherapies and pharmacotherapies) are needed to tailor to the treatment needs of older women.

Despite promising results that women with heavy alcohol consumption are transitioning to non-excessive drinking, current results also replicated that excessive drinking patterns are growing among older women, as has been observed in other national samples [2]. This data also demonstrates that as younger generations age, these women may be at increased risk for excessive alcohol use, in that being older at baseline in the present study was associated with a decreased likelihood of transitioning to excessive drinking. These results provide important background to explore hypotheses as to why such increases in drinking may be taking place. One promising avenue of investigation is the further exploration of the role of cognitive decline, as well as neurodegeneration in this later onset of excessive drinking among women. Overall, heavy drinking has been shown to leave women vulnerable to neurocognitive degeneration and memory deficits [4]. Additionally, emerging research suggests that microglia, macrophages involved in neuroinflammation, are excessively activated in response to alcohol, thus may result in alcohol-induced neurodegeneration. Alcohol-induced neurodegeneration has been closely linked to decreased cognitive functioning and poorer AUD treatment outcomes. This is concerning, as women may be more vulnerable to such neurodegeneration and cognitive decline, which may be related to increased alcohol consumption [for review, see 7]. However, additional research is needed to elucidate the reasons behind such behavioral changes among these women.

Results also warrant further exploration into the role of “reactive” drinking among older women. Previous research has demonstrated that alcohol consumption among women may increase as they age as a result of various psychosocial changes, such as retirement and widowhood [13]. Because women may be at heightened risk for using alcohol to regulate negative affect [7], women across the menopausal transition may be at higher risk for drinking in response to divorce, children leaving the home, retirement, illness, and isolation [4, 13]. The present study demonstrated that women who did not previously engage in excessive drinking are more likely to transition to excessive drinking as they progress through the stages of the menopausal transition. Given that women are faced with unique life stressors as they transition across menopause, future research should be conducted to establish if psychosocial events related to development are associated with increased alcohol consumption. Additionally, future studies should explore additional contributing factors to increase in alcohol consumption. For instance, while outside the scope of the present study, given that there is substantial evidence that androgens (e.g., testosterone) drive female libido and may be supplemented in postmenopause to treat sexual dysfunction [23], future inquiries regarding the association between testosterone, sexual activity, and drinking may be explored utilizing the SWAN dataset.

## Limitations

While the present study contributes to the scant literature exploring the changes in alcohol consumption across the menopausal transition, there are additional limitations that warrant mention. First, data included in these analyses included variables (e.g., sex hormone levels and depression) that were evaluated at only one time point annually. Additionally, the menopausal status categorization did not rely on biological confirmation. Thus, further studies with confirmatory biological measures are warranted. The present publicly available data did not include annual measures of progesterone, thus future studies should explore the role of progesterone fluctuations within these transitions. The publicly available datasets also did not include more in-depth variables to explore psychiatric diagnoses and specific alcohol-related variables (e.g., craving and withdrawal symptoms). Future studies would be strengthened by including such variables. In spite of such limitations, the present study contributes to the current understanding of transitions in alcohol use across menopausal stages.

## Perspectives and significance

The present study demonstrates that the menopausal transition is a period of instability and change regarding excessive drinking behaviors. Women may newly transition to excessive alcohol consumption during the menopausal transition, and other women who have existing excessive alcohol consumption may cease to be excessive drinkers. These results indicate the need for additional research to explore how factors may contribute to changes in alcohol consumption among women.

## Data Availability

The original datasets utilized in the current manuscript are achieved within the Inter-university Consortium for Political and Social Research (ICPSR) and available at https://www.icpsr.umich.edu/icpsrweb/ICPSR/series/00253.
